# Willingness to Work and the Working Environment of Japanese Dental Hygienists

**DOI:** 10.1155/2018/2727193

**Published:** 2018-08-26

**Authors:** Yoshiaki Nomura, Ayako Okada, Jun Miyoshi, Masaru Mukaida, Eriko Akasaka, Keietsu Saigo, Hideki Daikoku, Hidenori Maekawa, Tamotsu Sato, Nobuhiro Hanada

**Affiliations:** ^1^Department of Translational Research, Tsurumi University School of Dental Medicine, 2-1-3 Tsurumi, Tsurumi-ku, Yokohama 230-8501, Japan; ^2^Iwate Dental Association, 2-5-25 Morioka-eki-Nishi-dori, Morioka 020-0045, Japan

## Abstract

Japanese dental hygienists' employment rate is low. The environment factors and daily job contents that contribute to willingness to work of Japanese dental hygienists and their structures were investigated. A cross-sectional survey was conducted using a self-administered postal questionnaire distributed for one thousand and twenty-three members of Japan Dental Hygienist Association registered in Iwate prefecture affiliation. Three items concerning willingness to work, satisfaction for the 9 items about working environment, anxiety for work, and 106 daily job contents were used for analysis. Structural equation modeling, decision analysis, and correspondence analysis were carried out. The present study found that working environment such as interpersonal relationship was more important than social environment such as salary for the regular employee of Japanese dental hygienist working at private dental office. However, salary was only the determinant for the dental hygienist who strongly disliked their work. And daily job contents affected the willingness to work. Especially, jobs concerned with prosthodontic treatments were of major concern. Improving the working environment and avoiding assignment of tasks that require lower level of skill may improve dental hygienists' willingness to work and may assist to improve the employment rate of dental hygienist in Japan.

## 1. Background

Dental hygienists must be competent and well qualified to be accepted by society [1]. The demand for dental hygienists has grown in recent decades, and their professional roles are continuously changing based on the national healthcare service system [2]. In Japan, dental hygienists provide a wide range of services [3, 4]. In response to the increased demand, the duration of dental hygiene education has been extended in Japan as well as in European countries [5, 6]. Additionally, topics concerning cooperation with other professionals, systemic diseases, and rehabilitation have been introduced into the educational curriculum [7, 8]. In the aging Japanese society, the visiting care support system has become prevalent, and the educational program described above supports the work of dental hygienists in this system. In fact, dental hygienists play an important role in the prevention of respiratory infections among elderly individuals through oral care [9]. However, the number of dental hygienists providing services in the visiting care support system remains insufficient.

The employment rate of dental hygienists in Japan is quite low [10] compared with those in other countries [11–13], as dental hygienists in Japan need to obtain a national license to work in the dental profession. Indeed, the number of licensed dental hygienists is estimated to be approximately 250,000, while the number of working dental hygienists is only 116,299 (2014) [14].

The reasons for this low employment rate are complex. Gorter [15] found that 13% of dental hygienists felt emotionally exhausted due to work stress, the need to balance their work life and private life, the long working hours, the lack of an assistant, and the lack of positive encouragement from employers. The factors that lead dental hygienists to leave clinical practice include increasing family responsibilities [16]; a lack of dental hygiene training and experience; employee benefits [17]; and a lack of respect, collaboration, and appreciation from employers [18]. According to a survey conducted by the Japanese dental hygienists' association, factors such as marriage, childbirth, childcare, a lack of ability, scope of practice, overwork, low wages, anxiety about employment, and working conditions are associated with the low employment rate of dental hygienists [19]. Thus, maintaining a healthy work-life balance, a healthy lifestyle, and a good work environment may contribute to dental hygienists' willingness to work. Additionally, improving the working environment may be important for dental hygienists and may also decrease their likelihood of leaving the profession. These challenges are intertwined, and their interactions add to the complexity of the low employment rate among dental hygienists.

In this study, we investigated specific environmental factors that contribute to the willingness to work among Japanese dental hygienists and their working conditions. In addition, we sought to identify the specific daily job contents that led to dissatisfaction among dental hygienists.

## 2. Study Population and Methodology

### 2.1. Study Population

This study was a cross-sectional survey conducted using a self-administered postal questionnaire. The questionnaires were distributed to one thousand and twenty-three dental hygienists living in Iwate Prefecture between 14 January and 27 January 2016. The deadline for responses was set to 9 February 2016. In 2015, the Japanese Dental Hygienist Law was amended so that males could be dental hygienists. Therefore, all respondents in the present study were females.

### 2.2. Questionnaire

The questionnaire consisted of 26 major categories including demographic factors, work content, motivation for work, working conditions, and company benefits. Among these items, the following were used for the analysis. Three items regarding dental hygienists' willingness to work were investigated using a five-point Likert scale, with responses including strongly agree, agree, disagree, strongly disagree, and neither. These three items consisted of “The work of a dental hygienist is worth doing,” “I like working as a dental hygienist,” and “The work of a dental hygienist is suitable for me.” Satisfaction regarding 9 items assessing the working environment was investigated using a four-point Likert scale, with responses including very satisfied, satisfied, dissatisfied, and very dissatisfied. These 9 items consisted of salary, anxiety about health and overwork, company benefits, long work hours, anxiety over employment, job description, interpersonal relationships with dentists, interpersonal relationships with other dental hygienists, and work competency. The daily job contents of a dental hygienist were assessed through 106 items regarding dental treatments and their role in assisting with these treatments.

### 2.3. Data Analysis

A factor analysis was carried out to extract the features that contribute to willingness to work using a maximum likelihood method with varimax rotation. Eigenvalues of 1 or more were extracted as factors. Then, structural equation modeling was performed to depict the structure of each constructive concept and each item. The root mean square error of approximation (RMSEA) was used for the fitness index [20]. To determine the characteristics of the minority subjects who answered strongly disagree for the items regarding willingness to work, a classification and regression tree (CART) analysis was conducted [21–23]. For the correlation of daily job contents with willingness to work, cross tabulations were constructed and chi-square tests were carried out. For the daily job contents showing statistically significant correlations with willingness to work, a correspondence analysis was performed. These analyses were conducted with IBM SPSS Statistics version 24 and IBM Amos 24 (IBM Inc., Tokyo, Japan).

### 2.4. Ethical Approval

The study was carried out in compliance with the principles of the Helsinki Declaration. Informed written consent for publication was obtained from each subject when the questionnaires were returned. The study was approved by the Ethics Committee of Tsurumi University, School of Dental Medicine (approval number: 430).

## 3. Results

One thousand and twenty-three questionnaires were distributed, and 502 were completed (the collection rate was 49.1%). Among the 502 responders, 449 dental hygienists were currently working, including 320 (63.7%) dental hygienists with regular employment at private dental offices (mean age: 38.6 ± 11.6 years), 97 (19.3%) with part-time employment at private dental offices (mean age: 43 ± 9.6 years), and 32 (6.4%) with other types of employment (mean age: 49.3 ± 12.0 years).

**Figure 1:**
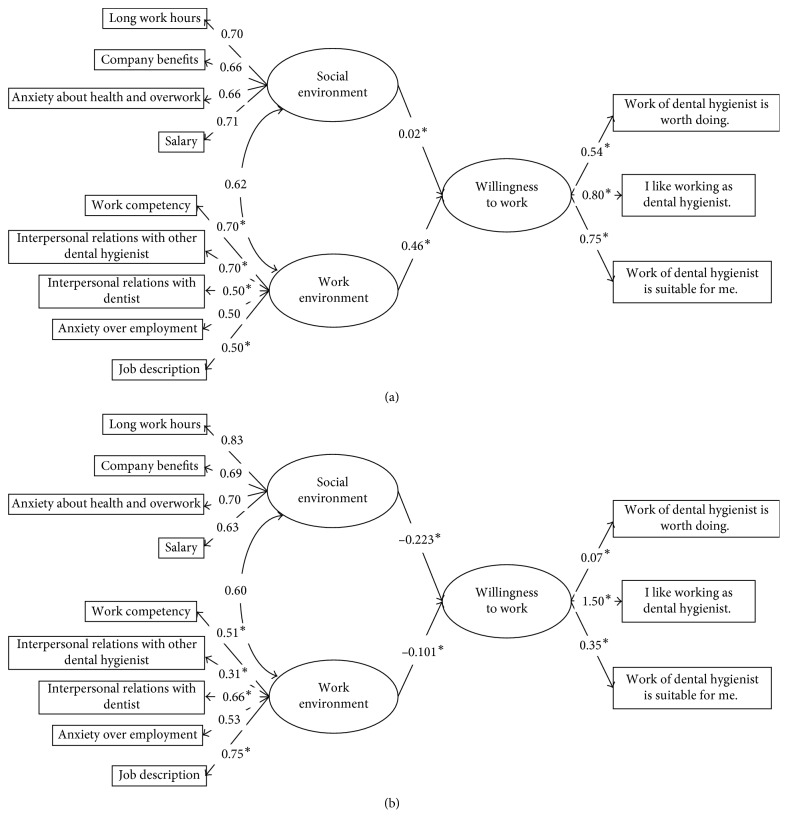
The path diagram of Japanese dental hygienists, their willingness to work at private dental offices, and their working and social environments. (a) Regular employees. (b) Part-time workers. All paths were statistically significant for the model of regular employment (a). In contrast, the paths from “Social environment” “to “Willingness to work” and “Work environment” to “Willingness to work” were not statistically significant for the model of part-time workers (b). ^*∗*^Statistically different paths between regular employees and part-time workers based on the multiple-group analysis.

In the factor analysis, the 9 items regarding satisfaction with the working environment were categorized into two factors. We termed these two factors “Social environment” and “Work environment.” Then, we carried out structural equation modeling. For the data from regular employees, all paths were statistically significant (Figure 1(a)). In contrast, for the part-time workers, the paths from “Social environment” to “Willingness to work” and from “Work environment” to “Willingness to work” were not statistically significant (Figure 1(b)). According to the multiple-group analysis, the following paths showed statistical differences between the regular employment group and the part-time job group: from “Social environment” to “Willingness to work”; “Work environment” to “Willingness to work”; “Willingness to work” to the three observed variables; and “Work environment” to “Work Competency,” “Interpersonal relationships with other dental hygienist,” “Interpersonal relationships with dentists,” and “Job description.”

**Figure 2:**
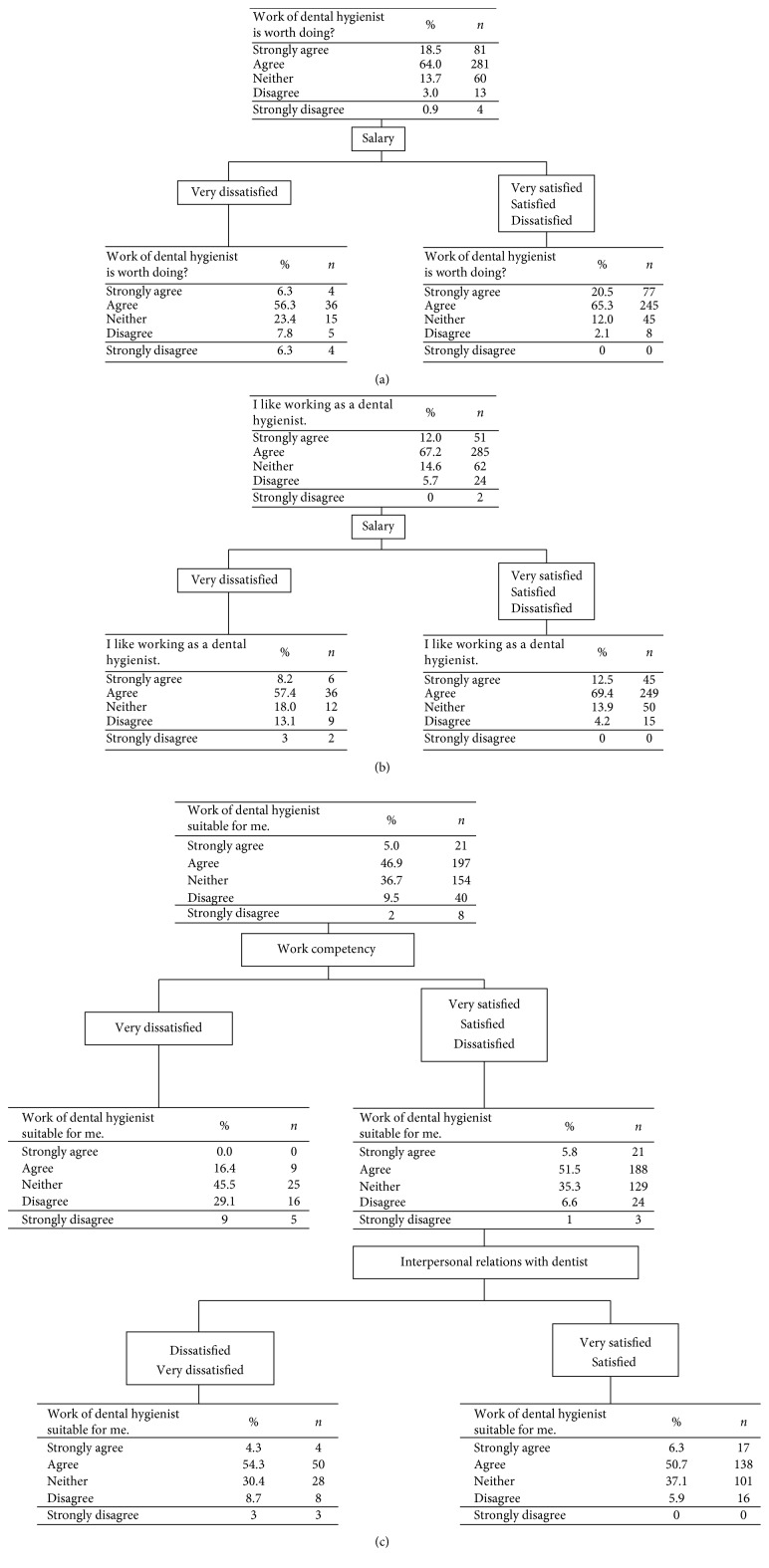
A decision tree to identify subjects who answered “Strongly disagree” for the item “The work of a dental hygienist is worth doing” (a), “I like working as a dental hygienist” (b), and “The work of a dental hygienist suits me” (c). For the items “The work of a dental hygienist is worth doing” and “I like working as a dental hygienist,” salary was the only determinant.

For the three items concerning willingness to work, very few subjects answered “Strongly disagree.” To further investigate these subjects, CART analysis was carried out. For the items “The work of a dental hygienist is worth doing” and “I like working as a dental hygienist,” only salary was a determinant (Figures 2(a) and 2(b)). The subjects who answered “Strongly disagree” for “The work of a dental hygienist is suitable for me” were very dissatisfied with “Work Competency” and were dissatisfied or very dissatisfied with “Interpersonal relationships with dentists” (Figure 2(c)).

The correlations between daily job contents and willingness to work were analyzed by chi-square tests. The item “The work of a dental hygienist is worth doing” showed statistically significant correlations with only two job contents: SPT and the presence of a removable occlusal guiding appliance. Statistically significant correlations were found between 14 job contents and the item of “I like working as a dental hygienist” and between 5 job contents and “The work of a dental hygienist is suitable for me.” For these two items, correspondence analyses were performed to summarize the cross tabulations. Additionally, the same procedures were carried out for the item “I feel annoyed by dental hygienist work.”

**Figure 3:**
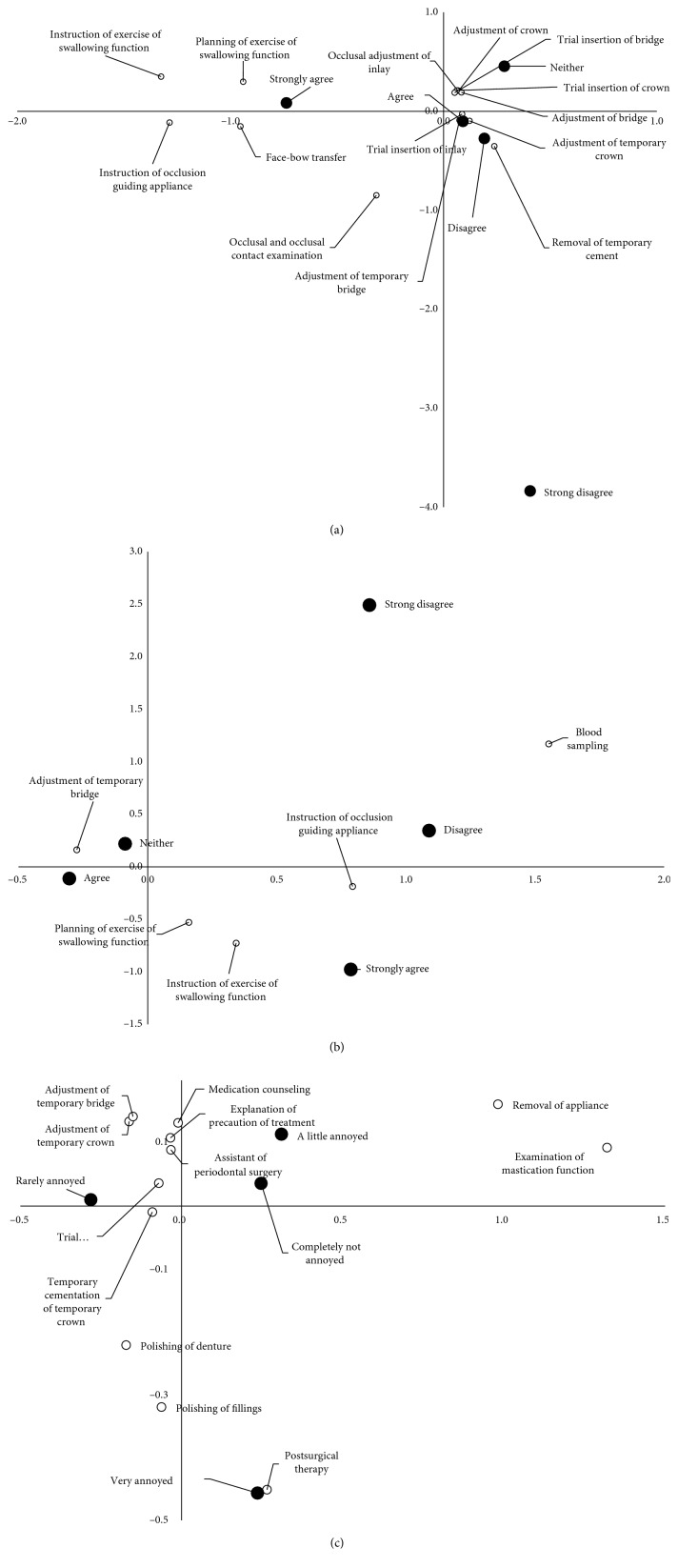
Biplot diagram for daily job contents and “I like working as a dental hygienist” (a), “The work of a dental hygienist suits me” (b), and “I feel annoyed by dental hygienist work” (c). Only job contents with statistically significant correlations with each question by chi-square tests were used for the correspondence analysis. As shown in (a) and (b), subjects who answered “Strongly disagree” were located far from the other points. Several job contents concerning prosthodontic treatments were scattered.

A biplot of the 14 job contents and “I like working as a dental hygienist” is shown in Figure 3(a). In this plot, swallowing function exercises and face-bow transfer were located near the dental hygienists who answered “Strongly agree.” The removal of temporary cement was located near the dental hygienists who answered “Disagree.” Job contents concerning prosthodontic treatments were located near the dental hygienists who answered “Agree,” “Neither,” and “Disagree.” Dental hygienists who answered “Strongly disagree” were located far from the other points.

For the item “The work of a dental hygienist is suitable for me,” the exercise of swallowing function was located between “Agree” and “Strongly agree.” Blood sampling was located far from the other job contents but between “Disagree” and “Strongly disagree.” “Strongly disagree” was located far from the other points (Figure 3(b)).

For the item “Annoyed with work,” “Very annoyed” was located far from the other points. Polishing of dentures, polishing of fillings, and postsurgical therapy were located near “Very annoyed.” Items concerning provisional restorations were located near “Little annoyed,” “Rarely annoyed,” and “Completely not annoyed” (Figure 3(c)).

## 4. Discussion

In this study, we evaluated the willingness to work among dental hygienists in Japan and its related factors. The social environment and the working environment were significantly related to the willingness to work among regular employees, although the correlation with the social environment was very weak. In contrast, among part-time workers, neither the social environment nor the working environment showed a statistically significant correlation with the willingness to work. Among regular employees, interpersonal relationships and job description may be important for a comfortable work environment.

The contributions of the factors that constitute willingness to work were very different between regular employees and part-time workers. Part-time workers placed little or no value on the job contents of dental hygienists, and they may not feel suitable for the job; however, they preferred the work of a dental hygienist more than that of regular employees. Thus, part-time workers seem to simply enjoy the work of a dental hygienist. This enjoyment may be one of the reasons that neither the social environment nor the working environment showed a statistically significant correlation with willingness to work in this group.

The motivating factors for dental hygienists included good relationships with coworkers, managers, and patients [18, 24–26]. When comparing the coefficients of part-time workers with those of regular employees, interpersonal relationships with other dental hygienists were less motivating, while interpersonal relationships with dentists were more motivating. In Japan, 57.5% of dentists are the coordinators of dental clinics, and most dental clinics are private [27]. Therefore, among part-time workers, interpersonal relationships with the coordinators may be more important than those with other colleagues. The mean age of the part-time workers was greater than that of the regular employees. Therefore, part-time workers may not care about their work competency, but they may be particular about the job description; the results presented in Figure 2(c) may support this conclusion.

For the subjects who answered “Strongly disagree” for “The work of a dental hygienist is worth doing” and “I like working as a dental hygienist,” only salary was a determinant. Several studies have reported that dental hygienists and therapists are dissatisfied with their remuneration package and the connection between their pay and their performance. Indeed, financial rewards play an important role in determining worker retention and job satisfaction [28]. Dental hygienists' and therapists' dissatisfaction with low remuneration have been reported in studies in the US [17], Australia [29, 30], Sweden [18], and South Africa [31]. The mean annual wage of a Japanese dental hygienist is ¥3,534,300 (approximately 32,000 US$), which is slightly higher than the mode of Japanese household income (¥2,500,000–¥3,000,000) but lower than the median (¥4,270,000) and the mean (¥5,419,000) [32]. In the US, the annual income of a dental hygienist is $73,440 [33]. Thus, the hourly wage is $15.8 for Japanese dental hygienists but $35.3 for US dental hygienists. US dental hygienists can coordinate their own clinics independent of dentists, and their job responsibilities are greater than those of Japanese dental hygienists. Nevertheless, the annual wage of Japanese dental hygienists is less than half of that of the US dental hygienists. One reason for this discrepancy may originate from the national insurance system of Japan; the Japanese insurance system covers a wide range of dental treatments, and the treatment fees are not expensive.

Several studies have shown that work tasks and variations in work are associated with job satisfaction and motivating factors among dental hygienists [13, 16, 18, 23]. The Japanese Dental Hygienist Law restricts the job range of dental hygienists to include the mechanical removal of dental plaque and dental calculi, the topical application of medicament on the tooth surface, dental treatment assistance, and health guidance. As shown in Figures 3(a) and 3(b), subjects who answered “Strongly disagree” were located far from the other points. These subjects may not be aware of the employment scheme. Dental hygienists with low levels of job satisfaction often report that the opportunities to practice their clinical skills are limited and that they are unable to fully utilize their abilities in the delivery of oral health services to the population [28]. The items concerning health instruction (i.e., planning and instructions for swallowing function) were located near the subjects who answered “Agree” and “Strongly agree.” Thus, the subjects who enjoy and value their job may provide health instructions. Working time other than oral health instruction, professional mechanical tooth cleaning, or scaling of supragingival calculi, dental hygienists in private dental offices need to work as dental assistants. Several job contents regarding prosthodontic treatments were scattered, although assistance with prosthodontic treatments may be part of the employment scheme for dental hygienists. Among these treatments, the removal of temporary cement was located near the subjects who disliked dental hygienist work. One of the contributing factors for low job satisfaction could be the unvarying assignment of tasks that require a significantly lower skill level than one's qualifications. For instance, the removal of temporary cement may be a monotonous task for the dental hygienist and may lead to job dissatisfaction.

Assistance in prosthodontic treatments may require advanced skills. As shown in Figure 3(c), dental hygienists who are annoyed with their work may be annoyed by the task of polishing dentures or fillings. Assistance in oral surgery may seem unconventional to dental hygienists. Indeed, blood sampling was located between feelings of being not suitable and strongly unsuitable as a dental hygienist, while postsurgical therapy was located near the dental hygienists who felt very annoyed with their work.

In summary, the present study found that the working environment, including interpersonal relationships, was generally more important than the social environment, including salary, among regularly employed Japanese dental hygienists working at private dental offices. However, salary was the only determinant for dental hygienists who strongly disliked their work. Additionally, job contents affected dental hygienists' willingness to work.

## 5. Conclusion

Identifying the factors associated with dental hygienists' willingness to work may help improve the employment rate of dental hygienists in Japan.
